# Feeding Mode Is Associated with Infant Night Sleep Trajectories During the First Postnatal Year

**DOI:** 10.3390/nu18111650

**Published:** 2026-05-22

**Authors:** Magdalena Olson, Li Liu, Elizabeth Reifsnider, Dean V. Coonrod, Sarada S. Panchanathan, Megan E. Petrov, Corrie M. Whisner

**Affiliations:** 1 College of Health Solutions, Arizona State University, Phoenix, AZ 85004, USA; mamezqu3@asu.edu (M.O.); liliu@asu.edu (L.L.); 2 Center for Health Through Microbiomes, The Biodesign Institute, Arizona State University, Tempe, AZ 85281, USA; 3 Biodesign Center for Personalized Diagnostics, Arizona State University, Tempe, AZ 85281, USA; 4 Ellmer School of Nursing, Old Dominion University, Norfolk, VA 23529, USA; ereifsni@odu.edu; 5 Department of Obstetrics and Gynecology, Valleywise Health, Phoenix, AZ 85008, USA; dean_coonrod@dmgaz.org; 6 College of Medicine, University of Arizona, Phoenix, AZ 85007, USA; 7 School of Medicine, Creighton University, Phoenix, AZ 85012, USA; 8 John Shufeldt School of Medicine and Medical Engineering, Arizona State University, Phoenix, AZ 85004, USA; 9 Department of Pediatrics, Valleywise Health, Phoenix, AZ 85008, USA; 10 Edson College of Nursing and Health Innovation, Arizona State University, Phoenix, AZ 85004, USA

**Keywords:** breastfeeding, formula feeding, mixed feeding, infant night sleep, dynamic feeding mode, night sleep trajectories, bedsharing, night-weaning, infant development

## Abstract

**Background:** Short sleep and formula feeding during infancy are associated with increased risk of childhood obesity. Feeding practices and sleep arrangements vary during infancy and may also be dynamic, yet their impact on infant night sleep duration remains unclear. Understanding these relationships is crucial for formulating recommendations to support breastfeeding and address sleep concerns. **Objective:** We examined the association between feeding mode and parent-reported infant night sleep duration during the first postnatal year, while additionally evaluating night-weaning and bedsharing as contextual sleep-related practices. **Methods:** Infants in the Phoenix Metropolitan Area (n = 193) were followed up at 3, 8, 13, 26, 39, and 52 weeks post-birth. Sleep and feeding questionnaires were answered at each visit. A multilevel growth model estimated infant night sleep duration trajectories by feeding mode (ordinal: exclusive formula, mixed, exclusive breastfeeding), night-weaning, and bedsharing as time-variant predictors. Maternal education and household income were covariates to account for differences in study attrition. **Results:** Infant night sleep duration followed a curvilinear trajectory, starting at 7.92 h (95% CI: 5.78, 10.06) and increasing by 0.40 h/month (95% CI: 0.21, 0.60), with a deceleration over time (0.02 h/month^2^, *p* < 0.001). Each increase in levels of breast milk consumption was associated with an increase in infant night sleep duration (B = 0.87 h, *p* < 0.001), but the association weakened as the infant aged (B = −0.07 h/month, *p* < 0.001). Despite 59.7% of bedsharing infants being exclusively breastfed, bedsharing was not significantly associated with infant night sleep duration. Similarly, night-weaning was not significantly associated with infant night sleep duration. **Conclusions:** Breastfeeding is associated with longer infant night sleep duration, whereas bedsharing showed no association despite its correlation with breastfeeding. This research highlights the importance of breastfeeding in early life, not only for its developmental benefits but also for its relationship with infant night sleep duration, an essential component of healthy infant growth.

## 1. Introduction

Childhood obesity is a growing concern and may begin early in infancy [1]. Among early obesity risk factors, early breastfeeding cessation and short sleep duration have shown consistent associations. For example, meta-analyses show a 17–26% decreased risk of obesity among breastfed infants compared to those who were formula-fed, and a dose–response effect by breastfeeding duration [2,3,4]. Similarly, a meta-analysis of 42 studies found that short sleep duration significantly increases the risk of overweight or obesity across all ages, including a 40% higher risk in infancy, 57% in early childhood, and 30% in adolescence [5]. The relationship of short sleep and obesity carries on into adulthood, where short sleep has been associated with increased weight gain and impaired energy metabolism [6]. Moreover, short sleep in infancy has also been related to emotional and behavioral problems later in life [7,8] and to reduced cognitive development at 2 years [9,10]. These findings suggest that the biological mechanisms linking sleep with multiple health outcomes may emerge early in infancy and persist throughout adulthood, highlighting the importance of establishing healthy sleeping habits early in life.

A newborn’s sleep–wake cycles are fragmented across the 24 h day, yet they gradually consolidate toward the night as circadian rhythms mature [11]. Feeding plays a central role in this process, as it provides nutrition and components that are also key regulators in infant sleep. Feeding at night is necessary early in life to support the high growth rate, and later on is still used to soothe infants during night awakenings [12]. Additionally, the type of feeding (i.e., exclusive breastfeeding, mixed feeding, or formula feeding) has been linked to nighttime sleep duration. Most studies report that breastfeeding is associated with longer night sleep [13,14,15], although not all report significant results [16], and other reviews noted mixed findings [17,18]. One explanation for longer sleep may lie in compositional differences that make formula slower to digest [19,20]. Moreover, formula feeding and, in many cases, pumped breast milk feeding lack the daily bioactive intake fluctuations found in breast milk that may support circadian rhythm synchronization [11,21,22]. Key breast milk components implicated in sleep regulation include melatonin [14,23], tryptophan [24], cortisol [25], and nucleotides such as 5’AMP, 5’GMP, and 5’UMP [22].

Concerns about infant and maternal sleep can influence parenting decisions such as feeding mode, bedsharing, and night feeding. Formula feeding is often perceived as a solution for reducing night awakenings because formula-fed infants generally wake less frequently and have fewer night feedings than breastfed infants [13,15]. Maternal sleep fragmentation is a common reason behind breastfeeding cessation [26]. Many mothers, motivated to continue breastfeeding, resort to bedsharing with their infant to minimize the maternal sleep disruption from nighttime feedings [27,28,29]. However, bedsharing has also been associated with more infant night awakenings [30,31,32] and shorter nighttime sleep duration [30], although these findings might be confounded by the mother’s availability and proximity [31]. Despite the American Academy of Pediatrics’ recommendation against it [33], bedsharing remains common, particularly among parents with perceived infant sleep problems [26,31]. Studies show that breastfeeding mothers are significantly more likely to bedshare, and this arrangement remains across the first year after birth [17,26,34].

As infants grow, parents may choose to night-wean, defined as the cessation of nighttime feeding, to address maternal and infant sleep concerns [17]. The timing of night-weaning could have important biological implications, as feeding at night may provide central and peripheral circadian cues through the breast milk’s fluctuating composition [11,21,22]. However, the effect of night-weaning timing on sleep and development remains an emerging area of research.

Although previous studies have shown that breastfed infants tend to sleep longer at night, most have relied on cross-sectional data of infant night sleep and/or feeding mode [13,14,15], and only a few accounted for other time-varying behaviors such as bedsharing [16,27]. To our knowledge, no study has evaluated the association between night-weaning and infant night sleep duration. This longitudinal study aims to integrate these dynamic behaviors and examine their relationships with infant night sleep during the first 12 months after birth using data from the Snuggle Bug|Acurrucadito study [35]. We hypothesized that greater breast milk consumption would be positively associated with parent-reported infant night sleep duration. We additionally explored whether bedsharing and night-weaning, two practices closely related to infant feeding and sleep, were associated with infant night sleep. Breastfeeding, bedsharing, and time to night-wean are highly personal decisions. A greater understanding of the dynamic relationships between feeding, sleep arrangements, and sleep could provide parents and health workers with information to facilitate decisions that best fit their unique family circumstances.

## 2. Materials and Methods

### 2.1. Participants and Recruitment

This study is a secondary analysis of the observational, longitudinal Snuggle Bug|Acurrucadito cohort conducted between November 2020 and May 2025 [35]. Healthy pregnant and postpartum women (18–40 y) with no alcohol or tobacco consumption during pregnancy and their infants with no growth or other developmental delay or abnormalities (n = 205) were recruited from the Phoenix Metropolitan Area, Arizona, USA. The mother-infant dyads were followed up for the first 12 months after birth with visits at 3, 8, 13, 26, 39, and 52 weeks. Study staff comprised promotoras, community health workers, and social workers. The Institutional Review Boards at Valleywise Health and Arizona State University approved the study protocol. For this analysis, we included 197 infants with data on night sleep, feeding mode, and night-weaning status at any time point.

### 2.2. Measures

Infant, maternal, and family demographics were collected during the first home visit, when the infant was 3 weeks old, via surveys. During each of the six visits, sleep, bedsharing, and night-weaning data were collected via the Brief Infant Sleep Questionnaire—Revised (BISQ-R), a validated parent-reported instrument [36]. Additionally, feeding mode variables were collected via the Centers for Disease Control and Prevention Infant Feeding Practices II Study Questionnaire. For the BISQ-R, participants reported on their infant’s sleep during the past two weeks, whereas for the feeding mode, they reported on the last 7 days.

### 2.3. Outcome

Infant night sleep duration was collected by asking the mothers: “How much total time does your child spend sleeping during the NIGHT (between when your child goes to bed and wakes for the day)?”

#### 2.3.1. Time-Variant Predictors

- Feeding mode was calculated based on breastfeeding and formula feeding status. If both were reported at a given visit, the infant would be considered mixed-fed during that visit. Feeding mode was modeled as an ordinal variable: exclusive formula feeding < mixed feeding < exclusive breastfeeding, representing increasing breastfeeding intensity. The encoding assumes equal spacing between categories and may not fully capture the heterogeneity within the mixed-feeding group. To address this, a sensitivity analysis treating Feeding Mode as categorical is included in the Supplementary Materials.
- Bedsharing status was defined by where the baby slept the majority of time: “Where does your child sleep for most of the night?” with response options of crib, own bed, parent’s bed, co-sleeper attached to parent bed, bassinet, swing, or other. A response of “parent’s bed” was assigned as positive for bedsharing status.
- Night-weaning status was defined by how the baby was put back to sleep during the night. Night-weaning was positive if participants did not select “Breastfeed/nurse my child back to sleep” nor “Bottle feed child back to sleep” to the question “When your child wakes up during the night, what do you do?”.

#### 2.3.2. Time-Invariant Predictor (Covariates)

Maternal education and household income, collected at baseline, were included as covariates because they were significantly associated with attrition in the full sample (n = 205), before applying exclusions for missing data, to avoid selection bias. Education was treated as an ordinal variable (No High School < High School or Technical Degree < Four-year Degree or more). Income was a categorical variable (High, Medium, Low, and Unknown) because participants could select “unknown” or “refuse to respond”.

### 2.4. Statistical Analysis

Demographic variables were compared between participants who withdrew from the study and those who completed it to determine variables to include as covariates in the final model. Categorical variables were analyzed with the Chi-Square Test (two-sided) or with Fisher’s exact test when the groups had <5 counts. Ordinal variables were tested with the Wilcoxon rank-sum test. Continuous variables were inspected for normality and analyzed using the t-tests and the Wilcoxon rank-sum test for normal and non-normal distributions, respectively. Brunner–Munzel tests were performed in cases of non-normality and heteroskedasticity. Statistical significance was established at a *p*-value lower than 0.05.

We used a multilevel growth model to analyze the effects of feeding mode, night-weaning, and bedsharing on infant night sleep duration over time. Although time is coded by study visit, the visits correspond closely to the infant’s age. Multilevel growth models use all available repeated observations under a missing-at-random (MAR) assumption, conditional on the observed variables included in the model. Maternal education and household income were included as covariates because they were associated with attrition.

Three models were fit sequentially: (1) an Unconditional Means Model, including random intercepts, to understand the variability of infant night sleep within and between infants; (2) an Unconditional Growth Model incorporating time as a fixed effect and random slope to model the increase in infant night sleep as they age and to account for the variability of the rate of that increase between infants, and (3) a predictor model to explore how the variables of interest are associated with infant night sleep over time. A quadratic time effect was included because the visual inspection of the longitudinal sleep trajectories suggested nonlinear growth and was confirmed by the improvement in model fit relative to the model with only the linear effect. All analyses were performed using R Studio (v. 2023.06.1+524) using the packages *lme4* (v. 1.1-37) [37] and *lmerTest* (v 3.1-3) [38]. Model fit was compared using the Akaike Information Criterion (AIC), the Bayesian Information Criterion (BIC), and an analysis of variance (ANOVA) test. The final model residuals were visually inspected for normality using Q-Q plots, homoscedasticity using residual vs. fitted plots, and the marginal and conditional R^2^ were calculated using the MuMIn package (v. 1.48.11) [39,40]. The predictor model was adjusted for additional sociodemographic covariates (sex, maternal age at delivery, birth weight, parity, cohabitation, race, ethnicity, and return to work; see Supplementary Materials).

## 3. Results

### 3.1. Descriptives

Of the 205 recruited mother-infant pairs, eight did not complete any surveys and dropped out after signing the consent. Four pairs had no visits with complete data. The final analytic cohort was composed of 193 pairs. The 193 participants contributed a total of 1800 person-months of follow-up, and the mean follow-up duration per participant was 9.3 ± 3.8 months. A total of 972 sleep measurements were obtained (mean 5.0 measurements per infant) and used for the three models. Differences among pairs who dropped out and those who remained in the study are presented in Table 1, and the participant flow diagram can be found in the Supplementary Materials Figure S1. Pairs who dropped out of the study had significantly lower maternal educational attainment and household income than those who completed it (Supplementary Materials Table S1).

### 3.2. Dynamics of Feeding Mode

**Figure 1:**
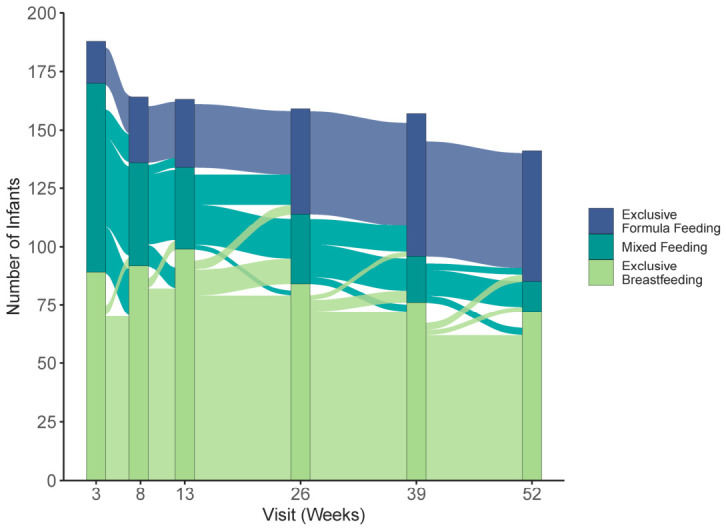
Waterfall plots of the dynamics in feeding mode by study visit. The total number of participants per visit was 188 (3 weeks), 164 (8 weeks), 163 (13 weeks), 159 (26 weeks), 157 (39 weeks), and 141 (52 weeks).

The shifts in infant feeding mode across infancy are shown in Figure 1 and summarized in Supplementary Materials Table S2. The prevalence of exclusive formula feeding increased with infant age, especially after 6 months, rising from 9.6% at 3 weeks to 39.7% at 52 weeks. The prevalence of mixed feeding decreased considerably, from 43.1% at 3 weeks to 9.2% at 52 weeks. Exclusive breastfeeding remained stable throughout the first year, with an overall prevalence of 52.7% across all visits. The cohort’s average breastfeeding duration was 8.3 ± 4.7 months. While some shifts between mixed feeding and exclusively breastfeeding occurred across infancy, once an infant began to exclusively formula-feed, they remained in that category.

### 3.3. Longitudinal Patterns of Night-Weaning and Bedsharing

**Figure 2:**
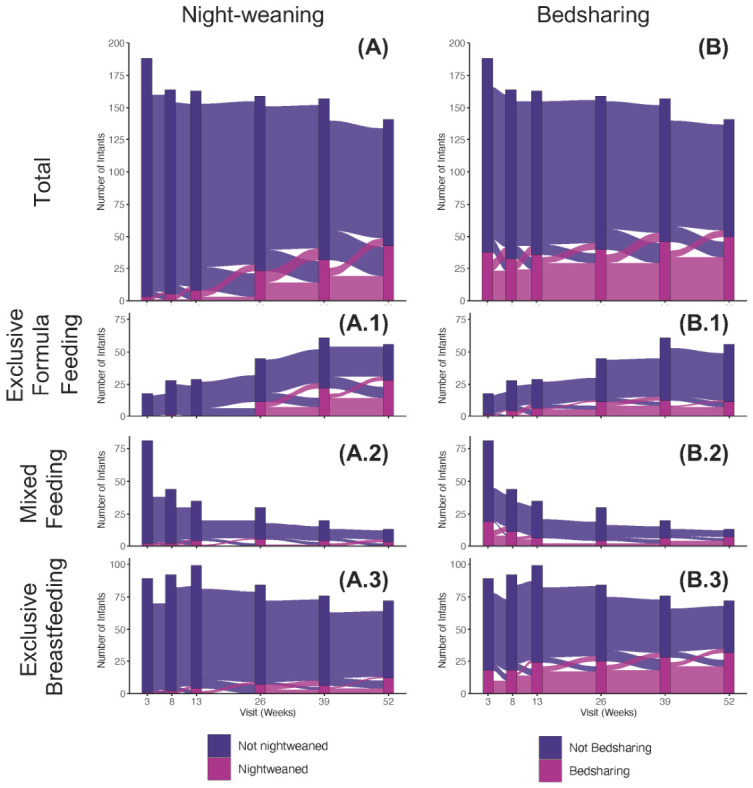
Dynamics of prevalence in (**A**) night-weaning and (**B**) bedsharing by feeding mode: Exclusive Formula Feeding (1), Mixed Feeding (2), and Exclusive breastfeeding (3). The bars represent the number of infants in each feeding mode at each visit. Because infants may change their feeding mode over time (e.g., from exclusive breastfeeding to mixed feeding), the bars do not necessarily represent the same infants across visits for these cases.

Night-weaning prevalence (depicted in Figure 2A) among exclusively formula-fed infants rose faster than the other two feeding groups after 6 months of age, from 24.4% to 50.0% at 12 months (Figure 2(A.1) and Supplementary Materials Table S2). The shifts between night-weaned and non-night-weaned groups indicate that night-weaning is not necessarily permanent, and parents may resume nighttime feeding (Figure 2(A.1–A.3)). Night-weaning prevalence differed by feeding mode (χ^2^(2) = 64.16, *p* < 0.001); formula-fed infants had a higher prevalence of night-weaning than exclusively breastfed and mixed-fed infants.

The prevalence of bedsharing is shown in Figure 2B and Supplementary Materials Table S2. Bedsharing occurrence increased by visit among exclusive formula-fed (from 5.6% to 19.6%) and exclusive breastfed infants (from 20.2% to 44.4%) (Figure 2(B.1,B.3)). Infants who were mixed-fed varied in bedsharing prevalence across the visits, with an overall prevalence of 23.8% (Figure 2(B.2)). Bedsharing prevalence among exclusively breastfed and mixed fed infants is higher than in the formula-fed group (χ^2^(2) = 7.762, *p* = 0.022). When accounting for all bedsharing occasions across the different time points rather than unique infants, 59.7% occurred while exclusively breastfeeding, 21.8% during mixed feeding, and 18.5% during exclusive formula feeding.

### 3.4. Feeding Mode, Night-Weaning, and Bedsharing Relationships with Night Sleep

**Figure 3:**
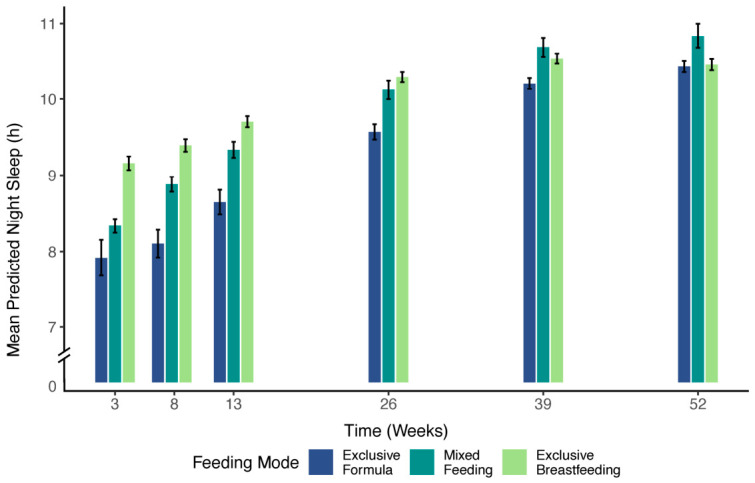
Predicted infant night sleep duration by feeding mode.

Infant night sleep duration increased from a mean of 8.6 ± 1.8 h at 0.7 months (n = 188) to 10.5 ± 1.2 h at 12 months (n = 141). The final multilevel growth model, shown in Table 2, incorporated all the predictors of interest. Infant age (time) was significantly associated with increased night sleep, 24.0 min/month (B = 0.400 h, *p* < 0.001), and a small negative effect of the quadratic time term that indicates that this nocturnal sleep duration growth rate decreases as the infant ages by 1.2 min/month^2^ (B = −0.02 h, *p* < 0.001). Feeding mode significantly predicted infant night sleep; each step towards breastfeeding (from exclusive formula feeding, to mixed feeding, and to exclusively breastfeeding) was associated with an additional 52.2 min of night sleep (B = 0.87 h, *p* < 0.001). This effect diminished with age by 4.2 min/month, as a negative interaction effect was also significant (B = −0.07, *p* < 0.01) (Figure 3). Neither night-weaning nor bedsharing was significantly associated with longer night sleep. Maternal education and household income were not associated with infant night sleep.

Interactions between bedsharing and feeding mode, night-weaning and time, and bedsharing with time were not significant and were removed to favor model parsimony. Sensitivity analyses yielded results that were consistent with the primary model (Supplementary Materials Tables S3–S5). Treating feeding mode as categorical rather than ordinal showed that both mixed feeding and exclusive breastfeeding were associated with longer infant night sleep relative to exclusive formula feeding, with the strongest association observed for exclusive breastfeeding. Additional adjustment for timing of solid food introduction (<6 months vs. ≥6 months) and for maternal and infant demographic variables did not alter the association between feeding mode and infant night sleep.

Although BIC increased slightly with the inclusion of additional predictors, model fit improved significantly in every model (Full Model χ^2^(11) = 55.0, *p* < 0.001), and the added variables were theoretically and empirically meaningful contributors to sleep duration. The fixed effects in our model explained approximately 26% of the variance in infant night sleep (marginal R^2^ = 0.26), and the total model, including subject-level random effects, explained ~55% (conditional R^2^ = 0.54). Feeding mode showed the strongest association with infant night sleep duration (semi-partial R^2^ = 0.037), followed by its interaction with time (semi-partial R^2^ = 0.019). The rest of the predictors contributed minimal unique variance (Bedsharing semi-partial R^2^ = 0.0001, Night-weaning semi-partial R^2^ = 0.0022) but may be involved in shared or interactive effects.

## 4. Discussion

Our study examined the relationships between infant night sleep with feeding mode, bedsharing, and night-weaning during the first year after birth, leveraging data from the longitudinal Snuggle Bug|Acurrucadito study [35]. Feeding mode was significantly related to nocturnal sleep duration across infancy. Higher levels of breast milk consumption were associated with longer infant night sleep duration, particularly in the first months after birth. Night-weaning was mostly prevalent among 6, 9, and 12-month-old formula-fed infants, while bedsharing was more prevalent among exclusively breastfed infants, but neither was associated with infant night sleep duration.

Feeding is a key *zeitgeber*—an external cue that controls our peripheral clocks—and may influence sleep regulation mechanisms [6]. Breast milk has been proposed to play a key role in circadian rhythm development, which may contribute to longer night sleep. In utero, infants’ rhythms are connected to their mother’s hormonal cycles, and after birth, this direct connection is lost, but breast milk may continue to provide maternal time cues through daily fluctuations in its composition of bioactive compounds (e.g., melatonin, cortisol) [11,21]. In contrast, formula-fed infants are fed the same composition over time and lack many of the bioactive components present in breast milk that relate to sleep–wake rhythms. Consistent with prior literature proposing these mechanisms, our data and previous reports show that breastfeeding is associated with longer night sleep duration [14,15,41]. We also observed that this effect attenuated over time, suggesting that feeding mode might be more strongly associated with infant night sleep before complementary feeding, when circadian rhythms are beginning to establish. This finding aligns with a study reporting that the mean difference in the effect of exclusive and predominant breastfeeding, relative to partial breastfeeding and exclusive formula feeding, on infant night sleep duration attenuates over time from 0.15 h at 3 months to 0.09 h at 12 months and no difference at 24 months [42]. In contrast, among older children (1- to 5-year-olds), breast milk intake shows an inverse relationship with night sleep duration [43]. The direction of the relationship may reverse in older children, who are receiving most (or all) of their nutrients from solid foods, potentially reducing the relative contribution of breast milk intake to sleep-related processes.

Breast milk contains melatonin, a hormone that neonates gradually begin to produce after birth [11,14], and its daily rhythmic secretion is not established until about 9–15 weeks [44]. Indeed, breastfed infants have shown more regularity in the increase in urinary melatonin metabolites and longer nocturnal sleep duration compared to formula-fed infants [14]. Besides providing direct melatonin, breast milk also contains tryptophan, a precursor to serotonin and subsequent melatonin synthesis [45,46]. Together, these findings suggest that the timing of bioactive nutrient exposure, rather than composition alone, might support sleep regulation, warranting further study.

Although breastfeeding may support circadian rhythm establishment, it is also linked to more night awakenings [18,47]. These awakenings, however, do not equate to shorter sleep, as breastfeeding has been associated with longer night sleep in the present study and previous studies [14,15,41]. However, it is unclear if the night awakenings are maladaptive. “Sleeping through the night” is a socially encouraged milestone, and is often assumed to be more common among formula-fed infants. However, it is highly variable between and within infants, and it does not imply the absence of night awakenings; infants may wake up and not require parental help to return to sleep [48]. Indeed, some authors have suggested that more night awakenings related to breastfeeding and parental proximity may protect infants who have trouble waking up on their own and who may be vulnerable to prolonged apneas [32]. Others have proposed that these awakenings help address the infant’s needs for frequent feeding and brain growth [17].

In our adjusted model, night-weaning was not a significant predictor of night sleep duration. The interpretation of this finding is challenging because night-weaning was mostly prevalent among formula-fed infants after six months of age. Therefore, any associations between night-weaning and night sleep may be difficult to distinguish from differences related to feeding mode. Formula may promote infant-driven night-weaning due to its composition and digestibility, but it lacks circadian bioactive components in breast milk [22,49]. Future studies, including both breastfed and formula-fed infants who night-wean, are needed to address whether night-weaning influences infant night sleep.

Similar to night-weaning, we found no significant association between bedsharing and infant night sleep. Our results contrast with those of Mindell et al. (2017), who reported that bedsharing was associated with shorter nighttime sleep [30]. A plausible explanation is that our models controlled for feeding mode, which may show a stronger association with night sleep than bedsharing. Consistent with prior literature, bedsharing remained strongly associated with breastfeeding [27,34], suggesting that bedsharing may be more closely related to infant feeding practices than to infant night sleep duration itself.

This study has multiple strengths, including its longitudinal design and the use of time-varying predictors. Most prior studies assessed breastfeeding duration or status at a given time; our study accounted for within-infant changes across the first year. Moreover, we have incorporated predictors commonly linked with feeding and sleep patterns (i.e., night-weaning, bedsharing) that may be relevant to circadian rhythm development, although circadian outcomes were not evaluated in the present analyses. The findings are generalizable to healthy term infants and applicable to U.S. feeding-style environments. The major limitations of this study include missing data due to dropouts, which is a common occurrence in longitudinal studies involving postpartum mothers. Infant night sleep duration was parent-reported, which may overestimate sleep relative to objective measures and may also vary according to feeding or sleep practices. However, parent-reported sleep measures are widely used in infant research and have shown high correlations with actigraphy and sleep diary measures in infants [36]. We could not capture the chronological variables required to examine the effects of breast milk composition, such as the time difference between expression and consumption of pumped milk. Our bedsharing variable captured the predominant infant sleep location but did not distinguish planned from reactive or partial-night from full-night bedsharing, nor did it capture sleep-safety context. Finally, mothers might have interpreted the night-weaning question in different ways; some might have fed their child and then performed another action to soothe them back to sleep, which would have been erroneously coded as positive for night-weaned infants. Moreover, we did not collect information about whether night-weaning was infant-driven or parent-driven to improve sleep. Additionally, it remains unclear if the biological implications of night-weaning differ by feeding mode, as most night-weaned infants were also formula-fed. Future studies should examine this, as it might signal digestive maturation.

## 5. Conclusions

Among the variables examined, feeding mode showed the strongest association with parent-reported infant night sleep duration, especially in the first six months. Plausible mechanisms may involve the breast milk bioactive components and circadian rhythm consolidation, although these pathways were not analyzed in the present study. Although night-weaning and bedsharing were hypothesized to be related to infant night sleep, neither emerged as a significant predictor in the adjusted models. Nonetheless, the prevalence of bedsharing among breastfeeding infants should be noted, as this practice might prolong breast milk exposure to infants and consequently improve their sleep. A deeper understanding of the mother’s rationale for bedsharing to support nighttime feeding and how to practice it safely is needed. Recommendations that acknowledge real-world behaviors and support breastfeeding women may be more effective than a one-size-fits-all message.

## Figures and Tables

**Table 1:** Participant demographics by study completion.

	Overall	Completed	Dropped	*p* Value
	n = 193	n = 159	n = 34	
Infant demographics				
Sex, female n (%)	107 (55.4)	89 (56.0)	18 (52.9)	
Ethnicity is Hispanic, n (%)	70 (36.3)	55 (34.6)	15 (44.1)	
Race, n (%)				
White	143 (74.1)	120 (75.5)	23 (67.6)	
Other or Multiracial	40 (20.7)	33 (20.8)	7 (20.6)	
Not reported	10 (5.2)	6 (3.8)	4 (11.8)	
Birth weight (kg), M (SD)	3.36 ± 0.32	3.36 ± 0.34	3.39 ± 0.25	
Birth weight z score, M (SD)	0.15 ± 0.69	0.14 ± 0.71	0.20 ± 0.54	
Delivery mode, n (%)				
Vaginal	152 (78.8)	122 (76.7)	30 (88.2)	
C-section	41 (21.2)	37 (23.3)	4 (11.8)	
Family Demographics				
Mother’s marital status, n (%)				
Married	151 (78.2)	127 (79.9)	24 (70.6)	
Unmarried/Cohabiting	28 (14.5)	20 (12.6)	8 (23.5)	
Single/Not Cohabiting	13 (6.7)	11 (6.9)	2 (5.9)	
Divorced/Separated	1 (0.5)	1 (0.6)	0 (0.0)	
Widowed	0 (0.0)	0 (0.0)	0 (0.0)	
Mother cohabiting, n (%)	179 (92.7)	147 (92.5)	32 (94.1)	
Mother’s education, n (%)				*
No High school	13 (6.7)	10 (6.3)	3 (8.8)	
High school or technical degree	58 (30.1)	42 (26.4)	16 (47.1)	
Four-year degree or more	122 (63.2)	107 (67.3)	15 (44.1)	
Household Income, n (%)				*
Low: <50,000 USD	36 (18.7)	28 (17.6)	8 (23.5)	
Medium: 50,000–100,000 USD	60 (31.1)	50 (31.4)	10 (29.4)	
High: >100,000 USD	78 (40.4)	70 (44.0)	8 (23.5)	
Unknown	19 (9.8)	11 (6.9)	8 (23.5)	
Parity, M (SD)	1.2 ± 1.3	1.1 ± 1.3	1.4 ± 1.4	
Household occupants, M (SD)	4.3 ± 1.6	4.3 ± 1.6	4.4 ± 1.4	
Mother born in the US, n (%)	145 (75.1)	121 (76.1)	24 (70.6)	
Mother’s time living in the US (%), M (SD)	85.5 ± 29.9	85.9 ± 29.8	83.9 ± 30.7	
Mother’s ethnicity is Hispanic, n (%)	70 (36.3)	55 (34.6)	15 (44.1)	
Return to work (mo), M (SD)	5.4 ± 4.0	5.5 ± 4.0	3.1 ± 2.5	

* *p* < 0.05.

**Table 2:** Multilevel growth model construction predicting infant night sleep by feeding mode, night-weaning status, and bedsharing status.

		UMM ^1^			UGM ^1^			FULL Model			
Random effect variance		Variance	SD		Variance	SD		Variance	SD		
Infant	Intercept	0.57	0.75		1.29	1.14		1.15	1.07		
	Time (mo)				0.01	0.10		0.01	0.10		
Residual		1.93	1.39		1.19	1.09		1.16	1.08		
Fixed effects		Estimate	SE	*p*	Estimate	SE	*p*	Estimate	SE	*p*	
Intercept		9.62	0.07	***	8.46	0.12	***	7.92	0.20	***	
Time (mo)					0.37	0.04	***	0.40	0.04	***	
Time quadratic (mo^2^)					−0.02	<0.01	***	−0.02	<0.01	***	
Feeding Mode ^2^								0.87	0.18	***	
Night-weaned ^3^								0.26	0.14		
Bedsharing ^4^								0.05	0.12		
Education ^2^								0.33	0.21		
Income (Medium) ^5^								0.23	0.21		
Income (High) ^5^								0.17	0.21		
Income (Unknown) ^5^								−0.04	0.29		
Time: Feeding Mode ^2^								−0.07	0.02	**	
Model fit											
df			3				7			18	
AIC			3572.1				3277.3			3244.3	
BIC			3586.7				3311.5			3332.2	
LL			−1783				−1631.7			−1604.2	
*χ*^2^ (df)							32.2 (1)	***		55.0 (11)	***

^1^ UMM: Unconditional Means Model; UGM: Unconditional Growth Model; mo: months; df: degrees of freedom; AIC: Akaike Information Criterion; BIC: Bayesian Information Criterion; LL: log-likelihood. ^2^ Linear effects of ordinal variable, not displaying quadratic values. ^3^ Relative to not night-weaned ^4^ Relative to not bedsharing ^5^ Relative to low income. ** *p* < 0.01, and *** *p* < 0.001.

## Data Availability

The original contributions presented in this study are included in the article/Supplementary Materials. Further inquiries can be directed to the corresponding authors.

## Supplementary Materials

The following supporting information can be downloaded at: https://www.mdpi.com/article/10.3390/nu18111650/s1. Figure S1: STROBE Participant Flow Diagram for Observational Studies; Table S1: Participant demographics by study completion and statistical test used for comparison; Table S2: Feeding mode by visit and within-feeding mode prevalence of night-weaned and bedsharing; Table S3: Full model comparing Feeding mode as ordinal and categorical; Table S4: Full model adjusting for age of starting solids; Table S5: Model and other covariates.

## Abbreviations

The following abbreviations are used in this manuscript:

AIC	Akaike Information Criterion
ANOVA	Analysis of Variance
BIC	Bayesian Information Criterion
BISQ-R	Brief Infant Sleep Questionnaire-Revised
