# Supplementary material for: Feeding Mode Is Associated with Infant Night Sleep Trajectories During the First Postnatal Year
Source: Nutrients. 2026 May 22;18(11):1650. doi: 10.3390/nu18111650 (PMC13257929; doi:10.3390/nu18111650)
Supplement: Supplementary file 1 [file nutrients-18-01650-s001.zip › Supplementary_Table_S1_Nutrients.pdf]

Feeding Mode Is Associated with Infant Night Sleep Trajectories During the First Postnatal Year - Magdalena Olson

Supplementary Table S1: Participant demographics by study completion and statistical test used for comparison.

	**Overall** n = 193	**Completed** n = 159	**Dropped** n = 34	***p* value**	**Test Results**
**Infant demographics**					
Sex, female n (%)	107 (55.4)	89 (56.0)	18 (52.9)		χ^2 = 0.018, df=1, p=0.894
Ethnicity is Hispanic, n (%)	70 (36.3)	55 (34.6)	15 (44.1)		χ^2 = 0.726, df=1, p=0.394
Race, n (%)					Fisher p=0.163
White	143 (74.1)	120 (75.5)	23 (67.6)		
Other or Multiracial	40 (20.7)	33 (20.8)	7 (20.6)		
Not reported	10 (5.2)	6 (3.8)	4 (11.8)		
Birth weight (kg), M (SD)	3.36 ± 0.32	3.36 ± 0.34	3.39 ± 0.25		W = 2701.0, p=0.996
Birth weight z score, M (SD)	0.15 ± 0.69	0.14 ± 0.71	0.20 ± 0.54		W = 2722.0, p=0.950
Delivery mode, n (%)					χ^2 = 1.582, df=1, p=0.208
Vaginal	152 (78.8)	122 (76.7)	30 (88.2)		
C-section	41 (21.2)	37 (23.3)	4 (11.8)		
**Family Demographics**					
Mother's marital status, n (%)					Fisher p=0.404
Married	151 (78.2)	127 (79.9)	24 (70.6)		
Unmarried/Cohabiting	28 (14.5)	20 (12.6)	8 (23.5)		
Single/Not Cohabiting	13 (6.7)	11 (6.9)	2 (5.9)		
Divorced/Separated	1 (0.5)	1 (0.6)	0 (0.0)		
Widowed	0 (0.0)	0 (0.0)	0 (0.0)		
Mother cohabiting, n (%)	179 (92.7)	147 (92.5)	32 (94.1)		
Mother's education, n (%)				*	W = 3312.5, p=0.015
No High school	13 (6.7)	10 (6.3)	3 (8.8)		
High school or technical degree	58 (30.1)	42 (26.4)	16 (47.1)		
Four-year degree or more	122 (63.2)	107 (67.3)	15 (44.1)		
Household Income, n (%)				*	Fisher p=0.013
Low: < 50,000 USD	36 (18.7)	28 (17.6)	8 (23.5)		
Medium: 50,000 - 100,000 USD	60 (31.1)	50 (31.4)	10 (29.4)		
High: > 100,000 USD	78 (40.4)	70 (44.0)	8 (23.5)		
Unknown	19 (9.8)	11 (6.9)	8 (23.5)		
Parity, M (SD)	1.2 ± 1.3	1.1 ± 1.3	1.4 ± 1.4		W = 2389.0, p=0.266
Household occupants, M (SD)	4.3 ± 1.6	4.3 ± 1.6	4.4 ± 1.4		W = 2381.0, p=0.260
Mother born in the US, n (%)	145 (75.1)	121 (76.1)	24 (70.6)		χ^2 = 0.208, df=1, p=0.648
Mother's time living in the US (%), M (SD)	85.5 ± 29.9	85.9 ± 29.8	83.9 ± 30.7		W = 2853.0, p=0.502
Mother's ethnicity is Hispanic, n (%)	70 (36.3)	55 (34.6)	15 (44.1)		χ^2 = 0.726, df=1, p=0.394
Return to work (mo), M (SD)	5.4 ± 4.0	5.5 ± 4.0	3.1 ± 2.5		W = 653.5, p=0.057

* p<0.05, ** p < 0.01, *** p<0.001
