# Supplementary material for: Feeding Mode Is Associated with Infant Night Sleep Trajectories During the First Postnatal Year
Source: Nutrients. 2026 May 22;18(11):1650. doi: 10.3390/nu18111650 (PMC13257929; doi:10.3390/nu18111650)
Supplement: Supplementary file 1 [file nutrients-18-01650-s001.zip › Supplementary_Table_S2_Nutrients.pdf]

Feeding Mode Is Associated with Infant Night Sleep Trajectories During the First Postnatal Year - Magdalena Olson

Supplementary Table S2: Feeding mode by visit and within-feeding mode prevalence of night-weaned and bedsharing.

**Visit (weeks)**		**Exclusive Breastfeeding**		**Mixed Feeding**		**Exclusive Formula Feeding**		**Total**		**p-value**
		**(n)**	**(%)**	**(n)**	**(%)**	**(n)**	**(%)**	**(n)**	**(%)**	
**3**	Total	89	47.3	81	43.1	18	9.6	188		
	Night-weaned	1	1.1	2	2.5	0	0.0	3	1.6	0.709
	Bedsharing	18	20.2	19	23.5	1	5.6	38	20.2	0.244
**8**	Total	92	56.1	44	26.8	28	17.1	164		
	Night-weaned	2	2.2	2	4.5	1	3.6	5	3.0	0.574
	Bedsharing	18	19.6	11	25.0	4	14.3	33	20.1	0.532
**13**	Total	99	60.7	35	21.5	29	17.8	163		
	Night-weaned	4	4.0	4	11.4	0	0.0	8	4.9	0.105
	Bedsharing	24	24.2	6	17.1	6	20.7	36	22.1	0.671
**26**	Total	84	52.8	30	18.9	45	28.3	159		
	Night-weaned	7	8.3	5	16.7	11	24.4	23	14.5	0.045
	Bedsharing	25	29.8	4	13.3	11	24.4	40	25.2	0.203
**39**	Total	76	48.4	20	12.7	61	38.9	157		
	Night-weaned	6	7.9	4	20.0	22	36.1	32	20.4	**<0.001**
	Bedsharing	28	36.8	6	30.0	12	19.7	46	29.3	0.090
**52**	Total	72	51.1	13	9.2	56	39.7	141		
	Night-weaned	12	16.7	3	23.1	28	50.0	43	30.5	**<0.001**
	Bedsharing	32	44.4	7	53.8	11	19.6	50	35.5	**0.004**
