# Supplementary material for: Feeding Mode Is Associated with Infant Night Sleep Trajectories During the First Postnatal Year
Source: Nutrients. 2026 May 22;18(11):1650. doi: 10.3390/nu18111650 (PMC13257929; doi:10.3390/nu18111650)
Supplement: Supplementary file 1 [file nutrients-18-01650-s001.zip › Supplementary_Table_S3_Nutrients.pdf]

Feeding Mode Is Associated with Infant Night Sleep Trajectories During the First Postnatal Year - Magdalena Olson

Supplementary Table S3: Full model comparing Feeding mode as ordinal and categorical

		FULL Model Feeding mode ordinal			Sensitivity Analysis Feeding mode categorical		
**Random effect variance**		**Variance**	**SD**		**Variance**	**SD**	
Infant	Intercept	1.15	1.07		1.15	1.07	
	Time (mo)	0.01	0.10		0.01	0.10	
Residual		1.16	1.08		1.16	1.08	
**Fixed effects**		**Estimate**	**Std. Error**	***p***	**Estimate**	**Std. Error**	***p***
Intercept		7.92	0.20	***	7.93	0.27	***
Time (mo)		0.40	0.04	***	0.43	0.05	***
Time quadratic (mo^2)		-0.02	<0.01	***	-0.02	<0.01	***
Feeding Mode[1]		0.87	0.18	***			
Mixed Feeding[2]					0.51	0.25	*
Exclusive Breastfeeding[2]					1.22	0.25	***
Night-weaned[3]		0.26	0.14		0.26	0.14	
Bedsharing[4]		0.05	0.12		0.05	0.12	
Education[1]		0.33	0.21		0.33	0.21	
Income (Medium)[5]		0.23	0.21		0.23	0.21	
Income (High)[5]		0.17	0.21		0.17	0.21	
Income (Unknown)[5]		-0.04	0.29		-0.04	0.29	
Time : Feeding Mode[1]		-0.07	0.02	**			
Time : Mixed Feeding[2]					0.00	0.04	
Time : Exclusive Breastfeeding[2]					-0.09	0.03	**
**Model fit**							
Number of infants			193			193	
Number of observations			972			972	
df			18			18	
AIC			3244.3			3244.3	
BIC			3332.2			3332.2	
LL			-1604.2			-1604.2	
$\chi 2$ (df)						No difference	

mo: months; df: degrees of freedom; AIC: Akaike Information Criterion; BIC: Bayesian Information Criterion; LL: log-likelihood.
[1]Linear effect of ordinal variable, not displaying quadrating values
[2]Relative to exclusive formula feeding
[3]Relative to not night weaned
[4]Relative to not bedsharing
[5]Relative to Low income; *** (p<0.001); ** (p=0.001); * (p=0.05)
* $p < 0.05$, ** $p < 0.01$, *** $p < 0.001$
