# Supplementary material for: Feeding Mode Is Associated with Infant Night Sleep Trajectories During the First Postnatal Year
Source: Nutrients. 2026 May 22;18(11):1650. doi: 10.3390/nu18111650 (PMC13257929; doi:10.3390/nu18111650)
Supplement: Supplementary file 1 [file nutrients-18-01650-s001.zip › Supplementary_Table_S4_Nutrients.pdf]

Feeding Mode Is Associated with Infant Night Sleep Trajectories During the First Postnatal Year - Magdalena Olson

Supplementary Table S4: Full model adjusting for age of starting solids.

	FULL Model			Model +Age of Solids Introduction			Model Restricted to Ages Before Starting Solids (<6 mo)		
**Random effect variance**	Variance	SD		Variance	SD		Variance	SD	
Infant Intercept	1.15	1.07		1.05	1.02		2.20	1.48	
Time (mo)	0.01	0.10		0.01	0.09		0.23	0.48	
Residual	1.16	1.08		1.16	1.08		1.16	1.08	
**Fixed effects**	Estimate	Std. Error	*p*	Estimate	Std. Error	*p*	Estimate	Std. Error	*p*
Intercept	7.92	0.20	***	8.06	0.27	***	7.40	0.49	***
Time (mo)	0.40	0.04	***	0.43	0.04	***	0.57	0.31	
Time quadratic (mo^2)	-0.02	0.00	***	-0.02	0.00	***	-0.04	0.08	
Feeding Mode[1]	0.87	0.18	***	0.99	0.18	***	1.16	0.34	***
Night-weaned[2]	0.26	0.14		-0.22	0.14		0.28	0.35	
Bedsharing[3]	0.05	0.12		<0.01	0.12		0.14	0.18	
Education[1]	0.33	0.21		0.24	0.22		0.19	0.28	
Income (Medium)[4]	0.23	0.21		0.29	0.23		0.40	0.27	
Income (High)[4]	0.17	0.21		0.19	0.23		0.14	0.27	
Income (Unknown)[4]	-0.04	0.29		-0.17	0.32		0.05	0.37	
Time : Feeding Mode[1]	-0.07	0.02	**	-0.08	0.02	***	-0.17	0.14	
Starting Solids ≥6 mo[5]				-0.05	0.14				
**Model fit**									
Number of infants		193			159			192	
Numer of observations		972			896			515	
df		18			19			18	
AIC		3244.3			3024.7			1867.4	
BIC		3332.2			3115.9			1943.8	
LL		-1604.2			-1493.4			-915.7	

mo: months; df: degrees of freedom; AIC: Akaike Information Criterion; BIC: Bayesian Information Criterion; LL: log-likelihood.
[1]Linear effect of ordinal variable, not displaying quadratic values
[2]Relative to not night weaned
[3]Relative to not bedsharing
[4]Relative to Low income
[5]Relative to <6 mo
* $p < 0.05$, ** $p < 0.01$, *** $p < 0.001$
