# Supplementary material for: Feeding Mode Is Associated with Infant Night Sleep Trajectories During the First Postnatal Year
Source: Nutrients. 2026 May 22;18(11):1650. doi: 10.3390/nu18111650 (PMC13257929; doi:10.3390/nu18111650)
Supplement: Supplementary file 1 [file nutrients-18-01650-s001.zip › Supplementary_Table_S5_Nutrients.pdf]

Feeding Mode Is Associated with Infant Night Sleep Trajectories During the First Postnatal Year - Magdalena Olson

Supplementary Table S5: Model and other covariates.

		FULL Model			FULL Model + Other covariates			FULL Model + Other covariates + Mother Return to Work		
**Random effect variance**		Variance	SD		Variance	SD		Variance	SD	
Infant	Intercept	1.15	1.07		1.10	1.05		1.06	1.03	
	Time (mo)	0.01	0.10		0.01	0.10		0.01	0.10	
Residual		1.16	1.08		1.16	1.08		1.14	1.07	
**Fixed effects**		Estimate	Std. Error	*p*	Estimate	Std. Error	*p*	Estimate	Std. Error	*p*
Intercept		7.92	0.20	***	8.04	0.25	***	8.08	0.30	***
Time (mo)		0.40	0.04	***	0.40	0.04	***	0.42	0.04	***
Time quadratic (mo^2)		-0.02	0.00	***	-0.02	<0.01	***	-0.02	<0.01	***
Feeding Mode[1]		0.87	0.18	***	0.81	0.18	***	0.82	0.19	***
Night-weaned[2]		0.26	0.14		0.26	0.14		0.32	0.14	*
Bedsharing[3]		0.05	0.12		0.08	0.12		0.13	0.12	
Education[1]		0.33	0.21		0.53	0.26	*	0.47	0.27	
Income (Medium)[4]		0.23	0.21		0.05	0.22		0.26	0.23	
Income (High)[4]		0.17	0.21		<0.01	0.22		0.20	0.24	
Income (Unknown)[4]		-0.04	0.29		0.06	0.29		-0.05	0.32	
Time : Feeding Mode[1]		-0.07	0.02	**	-0.07	0.02	**	-0.07	0.02	**
Mother's age at delivery[5]					0.02	0.02		0.03	0.02	
Birth weight[5]					-0.02	0.20		<0.01	0.21	
Parity[5]					0.08	0.07		0.03	0.07	
Mother Not Cohabitating[6]					-0.52	0.26	*	-0.56	0.29	
Infant ethnicity (Hispanic)[7]					-0.11	0.16		-0.27	0.17	
Infant Sex (Female)					0.16	0.13		0.25	0.14	
Infant Race (Not white)[8]					-0.35	0.17	*	-0.28	0.18	
Infant Race (Unknown)[8]					0.03	0.36		0.15	0.38	
Maternal return to work[5]								0.06	0.02	**
**Model fit**										
Number of infants			193			193			156	
Numer of observations			972			972			879	
df			18			26			27	
AIC			3244.3			3244.1			2987.4	
BIC			3332.2			3370.9			3116.4	
LL			-1604.2			-1596			-1466.7	
$\chi2$ (df)						16.275(8)	*		Different dimension	

mo: months; df: degrees of freedom; AIC: Akaike Information Criterion; BIC: Bayesian Information Criterion; LL: log-likelihood.
[1]Linear effect of ordinal variable, not displaying quadratic values
[2]Relative to not night weaned
[3]Relative to not bedsharing
[4]Relative to Low income
[5]Grand mean centered
[6]Relative to cohabitating
[7]Relative to not hispanic
[8]Relative to white
*Return to work model failed to converge at 0.012 (tol = 0.002)
* $p < 0.05$, ** $p < 0.01$, *** $p < 0.001$
