# Supplementary figures and images for: Feeding Mode Is Associated with Infant Night Sleep Trajectories During the First Postnatal Year
Source: Nutrients. 2026 May 22;18(11):1650. doi: 10.3390/nu18111650 (PMC13257929; doi:10.3390/nu18111650)

## STROBE Participant Flow Diagram for Observational Studies

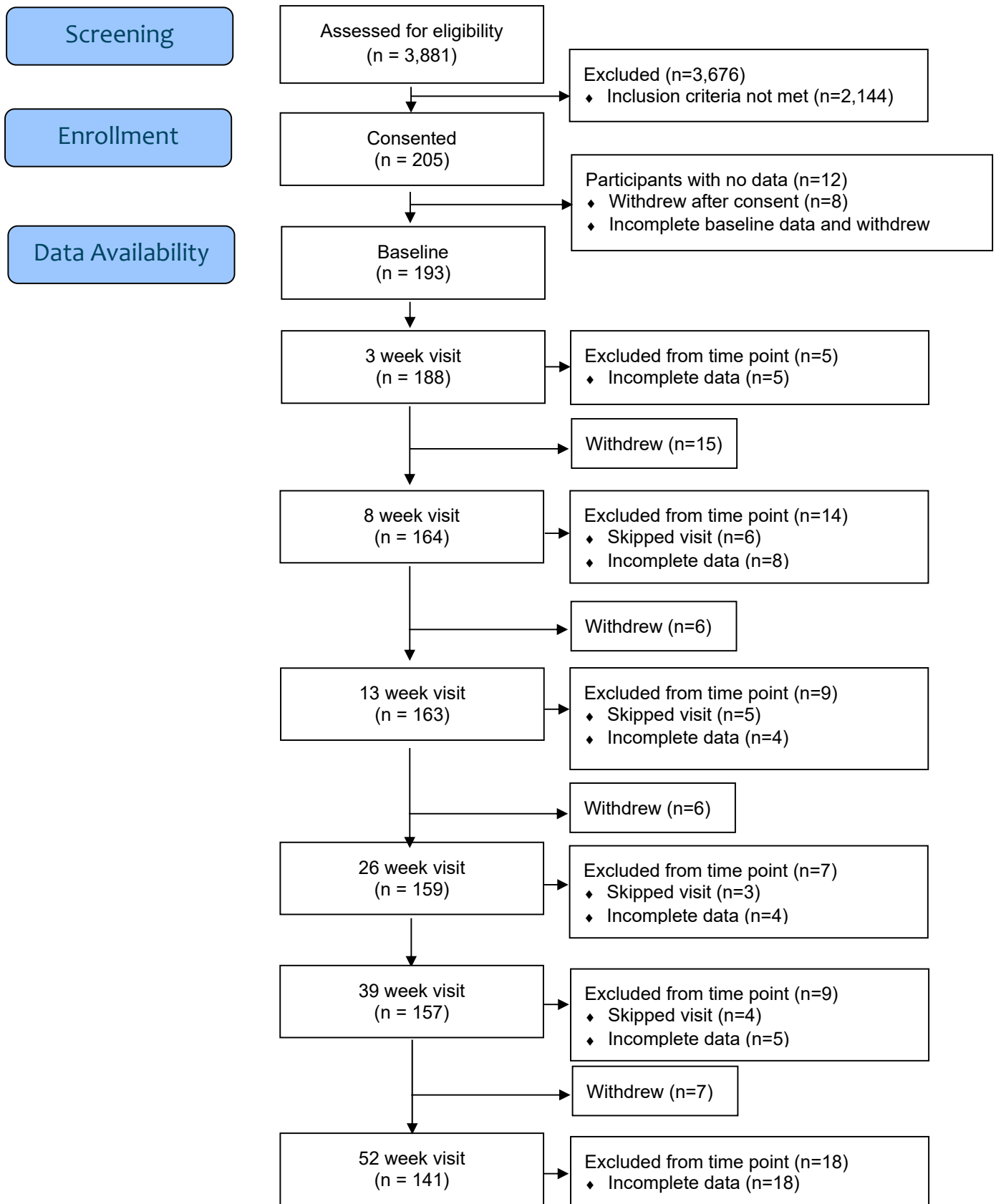

Supplement: Supplementary file 1 [file nutrients-18-01650-s001.zip › Supplementary_Figure_S1_STROBE_Participant_Flow_Diagram_Nutrients.pdf]
